# Exploiting monocarboxylate transporters as new molecular targets for colorectal carcinoma therapy

**DOI:** 10.1186/1753-6561-7-S2-P60

**Published:** 2013-04-04

**Authors:** Ricardo Amorim, Sandra Martins, Adhemar Longatto-Filho, Ana Preto, Fátima Baltazar

**Affiliations:** 1 Life and Health Sciences Research Institute (ICVS), School of Health Sciences, University of Minho, Campus de Gualtar, Braga, Portugal; 2 ICVS/3B’s – PT Government Associate Laboratory, Braga/Guimarães, Portugal; 3 Laboratory of Medical Investigation - LIM 14, Faculty of Medicine, São Paulo State University, São Paulo, Brazil; 4 Centre of Molecular and Environmental Biology (CBMA)/Department of Biology, University of Minho, Braga, Portugal

## Background

Tumour cells relies mostly on glycolysis to meet their energetic demands thus leading to an overload of lactic acid, which must be exported to the extracellular milieu, through the monocarboxylate transporters (MCTs). Due to their upregulation in cancer, MCTs are currently seen as promising therapeutic targets in cancer. Colorectal carcinoma (CRC) is the second most common type of cancer worldwide, being mainly a disease of industrialized countries. Among the chemotherapeutic agents used in the treatment of CRC, 5-fluorouracil (5-FU) is one of the most efficient, although resistance to 5-FU treatment has been reported. We aimed at understanding the role of MCTs in the glycolytic metabolism of CRC and exploring these transporters as putative therapeutic targets.

## Patients and methods

We performed a detailed characterization of MCT expression in a CRC clinical series and correlated the expression with key metabolic proteins. We also studied the effects of MCT activity inhibition in normal and CRC derived cell lines and evaluated the effect of MCT inhibitors in combination with 5-FU.

## Results

Our results showed that MCT1 and MCT4, their putative chaperones (CD147 and CD44) and the glucose transporter GLUT1 are overexpressed in CRC samples. Furthermore, we observed that MCT expression is associated with the remaining proteins. In *in vitro* assays, we demonstrated that MCT inhibition disrupted tumour cell aggressiveness and potentiated the cytotoxic effect of 5-FU.

## Conclusions

Our results provide novel evidence for the pivotal role of MCTs in CRC maintenance, supporting the exploitation of MCTs as therapeutic targets in CRC.

## Financial support

Portuguese Foundation for Science and Technology (FCT). The NCM460 cell line was received by a licensing agreement with INCELL Corporation, San Antonio, TX. The cells were routinely propagated under standard conditions in M3:10™ medium (INCELL).

